# Obesity and nutrition behaviours in Western and Palestinian outpatients with severe mental illness

**DOI:** 10.1186/1471-244X-11-159

**Published:** 2011-10-04

**Authors:** David Jakabek, Frances Quirk, Martin Driessen, Yousef Aljeesh, Bernhard T Baune

**Affiliations:** 1 Department of Psychology, James Cook University, Townsville, 4811, Australia; 2 School of Medicine and Dentistry, James Cook University, Townsville, 4811, Australia; 3 Department of Psychiatry and Psychotherapy Bethel, Ev. Hospital Bielefeld, Bielefeld, Germany; 4 Faculty of Nursing, Islamic University, Gaza, Palestinian Authority; 5 Discipline of Psychiatry, University of Adelaide, Adelaide, 5005, Australia

## Abstract

**Background:**

While people with severe mental illness have been found to be more overweight and obese in Western nations, it is unknown to what extent this occurs in Middle Eastern nations and which eating behaviours contribute to obesity in Middle Eastern nations.

**Method:**

A total of 665 responses were obtained from patients with serious mental illness attending out-patient clinics in Western developed countries (Germany, UK and Australia; n = 518) and Palestine (n = 147). Patients were evaluated by ICD-10 clinical diagnosis, anthropometric measurements and completed a self-report measure of frequencies of consuming different food items and reasons for eating. Nutritional habits were compared against a Western normative group.

**Results:**

More participants from Palestine were overweight or obese (62%) compared to Western countries (47%). In the Western sample, obese patients reported consuming more low-fat products (OR 2.54, 95% CI 1.02-6.33) but also greater eating due to negative emotions (OR 1.84, 95% CI 1.31-2.60) than patients with a healthy body-mass index. In contrast, obese patients from Palestine reported increased consumption of unhealthy snacks (OR 3.73 95% CI 1.16-12.00).

**Conclusion:**

Patients with mental illness have poorer nutritional habits than the general population, particularly in Western nations. Separate interventions to improve nutritional habits and reduce obesity are warranted between Western nations and Palestine.

## Background

People with severe mental illness (SMI) have been shown to be more overweight (body mass index [BMI] ≥ 25), obese (BMI ≥ 30), and have poorer nutritional status than the general population [1,2]. As a result of these levels of obesity, people with psychiatric disorders are at a greater risk of diabetes, cancer, cardiovascular and respiratory diseases [3-6]. With an increase in obesity across both developing and developed countries [7] the health consequences for people with a psychiatric illness increases the likelihood of negative outcomes and adds to the burden of disease.

The majority of research between SMI and obesity has primarily focused on people in Western nations and thus there is limited evidence to indicate whether these results can generalise to Middle Eastern nations. Associations between obesity and depression were found only in studies conducted in the United States [8], whilst a recent review found studies investigating the association between anxiety and obesity to be limited to Western countries [9]. In the case of schizophrenia, only male patients were found to have a statistically significantly lower BMI than a normative group in Iran [10] which is contrary to Western findings. Obesity is a growing concern among the general population worldwide due to an adoption of Western dietary patterns [11], however the impact of this on people with different mental illness in Middle Eastern nations is unclear.

Similarly, differences in eating behaviours between Western and non-Western patients with SMI has also received limited attention. Research using a broad range of Western and non-Western nations has argued that an increased consumption of sugar is associated with poorer outcomes in schizophrenia and increased prevalence of depression worldwide [12]. However, this study utilised population-level nutritional data which may not be representative of individual differences [13]. Middle Eastern individual-level investigations of nutrition and SMI are limited, focusing only on product consumption [10] and not eating behaviours such as frequency and reasons for eating. To the author's knowledge there are no English-language studies comparing nutritional behaviours of patients with SMI using consistent individual-level measures across Western and non-Western countries.

Lastly, the relative contribution of types of mental illness and eating habits towards body mass are infrequent in the literature. A comparison between mood, anxiety, schizophrenia and bipolar disorders was undertaken by Kilian et al. [14] involving psychiatric in-patients, however, patients living in the community are of significant interest as their nutritional status reflects the usual eating habits in a natural environment. In addition, community dwelling patients are more in control of their own eating habits.

The current study examines the relationship between BMI and nutritional habits of outpatients with primary psychiatric disorders or primary substance use disorders in three Western countries (Australia, Germany, UK) compared to one Middle Eastern country (Palestine) and a Western normative sample. It is hypothesised that both the Palestinian and Western groups will report poorer eating habits than the normative group. Moreover, it is hypothesised that the Palestinian patient group will report similar eating habits and BMI to the Western countries due to the effects of Westernisation of diet in Arabic countries such as Palestine. Finally, it is hypothesised that being overweight or obese is associated with increased consumption of unhealthy food groups and unhealthy eating habits across locations and psychiatric illnesses.

## Methods

### Sample

Participants were community-living patients from various outpatient clinics in Australia, Germany, UK, and Palestine. Patients gave informed consent to participate on intake into the clinics from 2001 to 2008 providing a response rate of 76.2% across all locations. They were classified based on ICD-10 F category diagnoses into seven disorder groups: Organic, substance use, schizophrenia and schizoaffective, depressive, neurotic and somatoform, behavioural and personality. Ethical approvals for this study were provided by the relevant local ethics committees.

### Assessment of BMI and nutrition

The participants' BMI was calculated by weight/height^2 ^(kg/m^2^). Weight and height were obtained by chart reports according to clinical measurements made by clinic staff. Four BMI categories were created: underweight (BMI < 18.5), normal weight (BMI = 18.5 - 24.9), overweight (BMI = 25 - 29.9) and obese (BMI > 30.0).

Dietary habits were assessed using the nutrition section of Dlugosch and Krieger's German-language General Health Behaviour Questionnaire [15]. This questionnaire was chosen as it includes a broad range of nutrition and eating behaviours and has demonstrated adequate validity and reliability in SMI samples. Certified translations of the questionnaire from German into English and Arabic languages were independently performed twice and the results were checked for inconsistencies. To enhance the validity of the instrument, an examination of face and content validity was conducted by submitting the questionnaire to experts. It was completed by patients on intake.

Dependant variables were calculated according to the General Health Behaviour Questionnaire manual, where ratings for separate food and drinking types were averaged to obtain mean frequencies of general dietary habits. Food consumption was measured on a 1 to 4 Likert scale with 1 indicating never performing the particular dietary habit and 4 indicating performing the dietary habit daily. The first composite measure was consuming healthy food and drinks, which contained items such as consumption of whole wheat bread, margarine, fresh vegetables, fruit, salad, and herbal tea. Other composite measures were: consumption of diet and low calorie products, such as margarine, low-fat milk and low-fat cheese; consumption of fast food, included eating burgers, chips and tinned meals; and finally eating traditional food, which included such items as sausages, eggs, cake, chocolate, beef, pork, coffee/black tea, and fruit juice. Regular eating habits reflected the frequency with which people reported eating breakfast, lunch, and dinner.

Eating habits were also examined by people responding to statements about eating behaviour on a 1 to 5 Likert scale with 1 representing "disagree" and 5 being "fully agree". Measures included frequency of unhealthy snacking (e.g. eating savoury items such as potato crisps between meals), eating food prepared outside the home, and eating in social situations. Finally, eating due to negative emotions was measured by how much respondents endorsed statements such as "I eat more than usual when I feel dejected/depressed" or "I eat more than usual when I am annoyed about something".

Summary normative data were provided from the General Health Behaviour Questionnaire manual. Normative data were not available for BMI or fast food consumption.

### Statistical analysis

Differences between groups were examined using Fisher's exact tests, independent Student t-tests and χ^2^-tests as appropriate. Comparisons between the normative sample with Palestinian and Western samples were performed using a two-way (location by diagnosis) ANOVA with post-hoc pair-wise comparisons using Bonferroni adjustments. Further analyses were conducted using multinomial logistic regression. In these subsequent analyses Palestinian patients in the underweight BMI category and substance abuse category were excluded due to low cell numbers (n = 2). Analyses were conducted using SPSS 19.0.

## Results

### Study participants

The sample consisted of 697 participants, 150 were from Palestine and 547 were from Western nations (Germany, n = 437; UK, n = 67; Australia, n = 43) Of these, 25 participants had primary diagnoses of organic, personality, or behavioural disorders, and were excluded from analyses. An additional 7 cases did not have a primary diagnoses recorded and so were also excluded from analyses. There were no statistically significant differences across demographic factors between excluded and included groups. Additionally, missing observations were noted in four cases for marital status and two cases for age; these missing values were imputed using multiple regression techniques based on available demographic information. BMI was unable to be calculated for 41 cases, so that these were analysed separately. No statistically significant differences of age, gender, education and marital status (p > 0.05) were observed between the groups where BMI was available and where BMI was missing; however, the groups differed in location (2% were missing in the Palestine group whilst 7% were missing in the Western group, Fisher's exact test = 0.02).

The available sample consisted of 665 participants with a mean age of 39.8 years (SD 11.4) ranging from age 15 to 78 years. Of the available sample forty-three (6.5%) patients were from Australia, 63 (9.5%) from the United Kingdom, and 412 (62%) from Germany. Sample characteristics between locations are presented in Table 1.

**Table 1:** Sample characteristics for Palestinian and Western groups

	Palestine n = 147	Western n = 518	p value*
Demographics (n, %)			
Age (M, SE)	35.94 (9.6)	40.92 (11.68)	<0.001
Male	88 (59.9)	289 (55.8)	0.397
Higher education	37 (25.2)	220 (42.5)	<0.001
Married	80 (54.4)	203 (39.2)	0.001
Disorders (n, %)			<0.001
Substance abuse	2 (1.4)	222 (42.9)	
Schizophrenia	88 (59.9)	70 (13.5)	
Depressive	49 (33.3)	171 (33)	
Neurotic and somatoform	8 (5.4)	55 (10.6)	
BMI category **, n (%)			0.023
Underweight	2 (1.4)	14 (2.9)	
Normal weight	54 (37.5)	240 (50)	
Overweight	57 (39.6)	155 (32.3)	
Obese	31 (21.5)	71 (14.8)	

* p value is for independent Student's t-test, Fisher's exact test or χ^2^-tests test as appropriate.

** For the Palestine group n = 144 and Western group n = 480.

Across the entire sample eating habits varied between demographic factors. Males reported greater consumption of traditional foods (p < 0.001) and fast food (p < 0.001) whilst females reported more frequent consumption of healthy food and drink (p < 0.001), diet products (p = 0.01) and eating due to negative emotions (p = 0.001). People with an A-level education or above reported more frequent eating due to negative emotions (p = 0.022). Participants who were married had a higher BMI (p = 0.003), ate more regular meals (p < 0.001), consumed less fast food (p = 0.05), snacked less (p = 0.03), and ate less food prepared outside the home (p = 0.02) than those who were not married. Means for eating habits across demographic factors are included in Table 2.

**Table 2:** BMI and nutritional behaviours across demographic categories

Health measure (M, SD)	Females	Males	Below A-levels	Above A- levels	Married	Unmarried
BMI	26.24 (6.05)	25.66 (5.41)	26.07 (5.50)	25.64 (5.98)	26.66 (5.77)	25.33 (5.57)
Healthy food & drinks	2.61 (0.41)	2.46 (0.40)	2.54 (0.40)	2.51 (0.42)	2.55 (0.37)	2.51 (0.43)
Diet products	2.35 (0.38)	2.27 (0.40)	2.29 (0.40)	2.32 (0.38)	2.32 (0.37)	2.29 (0.41)
Traditional products	2.50 (0.34)	2.62 (0.32)	2.57 (0.33)	2.58 (0.32)	2.59 (0.32)	2.56 (0.33)
Fast food	2.05 (0.49)	2.19 (0.50)	2.12 (0.48)	2.15 (0.54)	2.08 (0.50)	2.16 (0.51)
Regular meals	3.54 (0.61)	3.54 (0.55)	3.53 (0.59)	3.56 (0.55)	3.65 (0.48)	3.46 (0.62)
Snacking	2.48 (0.86)	2.53 (0.73)	2.51 (0.81)	2.52 (0.76)	2.44 (0.77)	2.57 (0.80)
Eating out	2.37 (0.78)	2.36 (0.83)	2.32 (0.81)	2.43 (0.78)	2.28 (0.78)	2.42 (0.81)
Emotional eating	2.21 (0.99)	1.96 (0.92)	1.99 (0.91)	2.18 (1.03)	2.01 (0.95)	2.11 (0.97)
Social eating	2.91 (0.92)	2.86 (0.86)	2.87 (0.87)	2.91 (0.92)	2.87 (0.93)	2.90 (0.86)

Means for consumption of products are on a 1 to 4 Likert scale and means for eating behaviours are on a 1 to 5 Likert scale.

Participants eating behaviours were compared across locations controlling for ICD-10 diagnosis and the results are presented in Table 3. In summary, the Palestinian group had increased consumption of all food types, more regular consumption of meals and higher unhealthy snacking than the Western clinical group. Compared to the normative group the Palestinian group also reported increased food consumption across all categories except for healthy food and drink. In contrast, the Western group reported less consumption of healthy food and drink and poorer eating habits than the normative group. Further comparisons were made between separate Western nations (Germany, Australia and UK) and the Palestinian and normative groups. No additional statistically significant differences were observed.

**Table 3:** Comparison of food consumption and eating behaviours between a German normative sample, Palestinian clinical and Western clinical countries

	Palestine n = 147	Western n = 518	Normative			
	**M (SD)**	**M (SD)**	**M (SD)**	**n**	**F-value**	**p**

Healthy food & drinks	2.75_a _(0.08)	2.52_ab _(0.02)	2.84_b _(0.01)	994	7.68	0.006
Diet products	2.48_ab _(0.08)	2.28_a _(0.02)	2.24_b _(0.01)	985	6.69	0.010
Traditional products	2.79_a _(0.70)	2.51_a _(0.02)	2.41_a _(0.01)	976	16.47	<0.001
Fast food	2.34_a _(0.10)	2.09_a _(0.03)			6.09	0.014
Regular meals	3.83_a _(0.10)	2.50_ab _(0.03)	3.72_b _(0.02)	1101	10.78	0.001
Unhealthy snacking	3.01_a _(0.15)	2.48_a _(0.04)	2.35_a _(0.02)	999	11.34	0.001
Eating away from home	2.18 (0.21)	2.46_a _(0.05)	2.20_a _(0.03)	1009	1.66	0.198
Emotional eating	1.99 (0.19)	2.19_a _(0.05)	1.83_a _(0.03)	996	1.04	0.309
Eating socially	2.59 (0.19)	2.88_a _(0.05)	3.05_a _(0.03)	1004	2.16	0.141

Means are estimated marginal means controlling for diagnosis type. Means for consumption of products are on a 1 to 4 Likert scale and means for eating behaviours are on a 1 to 5 Likert scale. Values with the same subscript differ at the p < 0.05 level in post-hoc tests with Bonferroni corrections. No normative information is available for fast food consumption.

Lastly, the risk patterns of different eating behaviours and disorders were examined for underweight, overweight and obese BMI categories relative to the normal BMI category. In the Western sample an increased consumption of diet products, reduced consumption of traditional foods and increase emotional eating was statistically significantly associated with obesity. Being underweight was statistically significantly associated with a lower consumption of healthy food and drinks. For the Palestinian sample, a different set of eating behaviours were associated with an increased BMI. Across both high BMI categories unhealthy snacking was identified as a risk factor, whilst lower consumption of diet products and infrequent eating in social situations was associated with a risk of being overweight. Across all locations there were no statistically significant risks for particular weight categories across different types of mental illness. ORs for eating behaviours and mental illness diagnostic categories across BMI categories controlling for demographic factors are provided in Table 4 for the Western sample and Table 5 for the Palestinian sample.

**Table 4:** Results for multinomial logistic regression comparing the association of eating habits and mental disorders between unhealthy BMI categories in Western countries

		Underweight n = 14			Overweight n = 155			Obese n = 71	
	**OR**	**95% CI**	**p**	**OR**	**95% CI**	**p**	**OR**	**95% CI**	**p**

Eating Habits									
Healthy food & drinks	0.07	0.01-0.53	0.010	1.11	0.59-2.09	0.743	0.85	0.36-2.00	0.717
Diet products	0.21	0.03-1.40	0.107	1.74	0.92-3.30	0.088	2.54	1.02-6.33	0.045
Traditional products	3.19	0.34-29.64	0.308	0.48	0.21-1.07	0.072	0.29	0.10-0.90	0.032
Fast food	2.38	0.66-8.51	0.184	1.42	0.85-2.37	0.175	1.80	0.89-3.65	0.105
Regular meals	1.27	0.45-3.54	0.654	0.89	0.60-1.31	0.559	1.16	0.66-2.03	0.608
Unhealthy snacking	1.04	0.39-2.77	0.939	1.17	0.83-1.64	0.362	1.34	0.85-2.10	0.209
Eating away from home	1.32	0.55-3.14	0.537	1.07	0.80-1.42	0.648	0.95	0.63-1.43	0.817
Emotional eating	0.82	0.41-1.66	0.590	1.18	0.91-1.55	0.217	1.85	1.31-2.60	<0.001
Eating socially	1.36	0.70-2.67	0.367	0.99	0.77-1.28	0.950	0.93	0.65-1.32	0.667
Disorder									
Schizophrenia		Reference							
Substance	0.30	0.02-5.49	0.417	0.58	0.27-1.23	0.153	0.70	0.21-2.37	0.569
Mood	1.07	0.07-15.57	0.960	0.83	0.38-1.79	0.628	1.45	0.44-4.75	0.542
Anxiety	5.46	0.38-79.1	0.214	0.96	0.36-2.54	0.927	3.54	0.98-2.76	0.054

Reference dependant category is healthy BMI. Adjusted OR's are reported controlling for demographic variables (age, gender, education and marital status) and Western locality (Germany, UK and Australia).

**Table 5:** Results for multinomial logistic regression comparing the association of eating habits and mental disorders between unhealthy BMI categories in Palestine

		Overweight n = 57			Obese n = 31	
	**OR**	**95% CI**	**p**	**OR**	**95% CI**	**p**

Eating habits						
Healthy food & drinks	1.85	0.22-15.58	.570	3.50	0.24-51.01	.360
Diet products	0.16	0.02-0.98	.047	0.71	0.08-6.28	.758
Traditional products	9.95	0.32-11.42	.191	0.05	0.00-8.86	.254
Fast food	0.40	0.1-1.54	.182	1.01	0.20-5.09	.994
Regular meals	0.12	0.01-1.73	.118	0.06	0.00-1.34	.076
Unhealthy snacking	2.60	1.15-5.87	.021	3.73	1.16-12.00	.027
Eating away from home	0.80	0.31-2.04	.635	0.72	0.24-2.16	.557
Emotional eating	1.67	0.65-4.27	.287	1.59	0.55-4.59	.388
Eating socially	0.34	0.14-0.81	.015	0.54	0.19-1.54	.248
Disorder						
Schizophrenia		Reference				
Mood	2.75	0.88-8.61	.083	2.91	0.81-10.42	.100
Anxiety	0.48	0.04-5.57	.553	4.01	0.55-29.31	.171

Reference dependant category is healthy BMI. Adjusted ORs are reported controlling for demographic variables (age, gender, education and marital status).

## Discussion

This cross-sectional study used the same instrument to examine nutrition in community-dwelling patients with different mental illnesses from Palestine and Western countries. Compared to the normative group, the Palestinian sample consumed more diet products, more traditional foods and consumed unhealthy snacks more frequently. A different set of eating behaviours were reported by the Western clinical sample, including fewer healthy food and drinks, fewer traditional products and more frequent eating due to negative emotions. Between the Palestinian and Western clinical samples, the Palestinian sample reported statistically significantly more frequent consumption of traditional foods and more frequent unhealthy snacking. Finally, these differences in eating behaviours were also associated with obesity across locations. In Palestine, more frequent unhealthy snacking was associated with a greater risk of being overweight or obese. In contrast, for Western patients with mental illness, a greater consumption of diet products and eating due to negative emotions were associated with obesity.

Unexpectedly, the Palestinian sample exceeded rates of obesity compared to the Western sample. For the Palestinian group, 40% of patients were overweight and 22% were obese, while the corresponding rates were 32% and 15% in the Western sample. This is in contrast to previous research which found that obesity in Middle Eastern nations should be approaching Western levels due to increasing urbanisation, changes in the availability and energy-content of food and reductions in physical activity [16,17]. Simultaneously, the results also indicate more frequent consumption of traditional foods and unhealthy snacking in the Palestinian group than the Western normative or clinical groups, which may be associated with the increased prevalence of obesity. In contrast, the Western clinical group consistently indicated less frequent consumption of healthy food and drink as well as greater eating due to negative emotions than the normative group. These differences in dietary composition indicate that Western-based studies examining nutritional composition of patients with SMI cannot directly be generalised to other non-Western countries.

The Western patients classified as obese had a 2.54 OR for consuming low-fat products and a 0.29 OR for eating traditional products (such as sausages, eggs, chocolate and cake which are typically high in fat). These results are inconsistent with previous literature which has found that people with SMI consume more high-fat meals [18] and less low-fat food types [19] than normative groups. This discrepancy in results may reflect obese patients actively working to consume a low-fat diet given the well known association between obesity and poor health outcomes. Unlike patients classified as obese, overweight patients ORs were only approaching statistical significance for both food types (p = .088 and .072, respectively). Consequently it is suggested that greater emphasis on interventions and encouragement to consume lower-fat products should be extended to patients in the overweight BMI range. Of further interest is the finding that Western patients who were obese reported a 1.85 OR of eating due to negative emotions and that this effect was not statistically significant for the Western overweight group or any Palestinian BMI group. Eating as an emotional regulation strategy is an extensive avenue of research [20] and may be a differentiating factor between overweight and obese patients with SMI. The results of this study suggest that in Western patients with SMI, nutritional interventions which specifically target eating due to negative emotions would be beneficial in reducing obesity in this population. However, this effect is location-specific and may not be relevant for Palestinian patients with SMI.

For the Palestinian sample the eating behaviours which contributed to being overweight and obese differed from the Western sample. Frequent snacking on unhealthy food items, such as eating potato crisps between meals, was the primary substantial risk factor for both being overweight and obese. Furthermore, low consumption of diet products and eating alone were also associated with an increased risk of being overweight. Thus in Palestinian patients with SMI, reducing frequent unhealthy eating would be an effective broad strategy to reduce BMI across weight categories.

There are some limitations with the present study that can be addressed by future research. Although patients were treated with medication appropriate to their disorder, thus making this study ecologically valid, specific types of medication were not taken into account. It has been demonstrated that certain medications are associated with changes in eating habits and body weight [21-23] and so future studies are encouraged to take into account medication types to better understand the proportion of variance medication contributes to differences in eating habits and weight. A second limitation is that the Palestinian and Western samples vary in demographic and diagnostic categories and so comparisons with the German normative group require some caution in attributing the differences in eating behaviours due to location alone. Nevertheless, demographic factors and diagnostic criteria are controlled for in logistic analyses; however the relatively low sample size in the underweight category limits the conclusions which can be made for this weight category. Further limiting conclusions is that a time lag is noted between the time of the normative data (published in 1995) and the data of the study (collected between 2001 and 2008). During this time BMI and negative eating habits have shown an upward trend [7], and so earlier results may underestimate the present association between mental illness, obesity, and eating habits. Furthermore, normative information is not available from Palestine, and thus regional differences in diet may be responsible for the observed differences in nutritional behaviours for people with SMI. Nevertheless, this does not detract from the conclusion that regional differences in patients with SMI should be taken into account when undertaking weight management interventions. Lastly, this study focused on only one Middle Eastern country (Palestine) and so the results are limited in their generalisability to other Middle Eastern populations.

## Conclusion

With the general move to community dwelling for people with mental illness, this study emphasises the need for monitoring and management of patients' diet in different ways depending on regional differences in nutrition detailed above. Further incorporation of nutritional management programs into outpatient management is encouraged to reduce subsequent disease and improve patients' quality of life.

## Competing interests

The authors declare that they have no competing interests.

## Authors' contributions

All authors have read and approved the final manuscript. DJ drafted the manuscript and conducted the statistical analysis, FQ provided critical editorial review, YA and MD were involved in data acquisition and revising the manuscript, BB conceived the study and provided extensive review of the manuscript.

## Pre-publication history

The pre-publication history for this paper can be accessed here:

http://www.biomedcentral.com/1471-244X/11/159/prepub
